# Inhibition of the NF-κB Signaling Pathway by a Novel Heterocyclic Curcumin Analogue

**DOI:** 10.3390/molecules20010863

**Published:** 2015-01-08

**Authors:** Anna-Maria Katsori, Ajay Palagani, Nadia Bougarne, Dimitra Hadjipavlou-Litina, Guy Haegeman, Wim Vanden Berghe

**Affiliations:** 1 Department of Pharmaceutical Chemistry, School of Pharmacy, Aristotle University of Thessaloniki, Thessaloniki 54124, Greece; E-Mails: akatsori@pharm.auth.gr (A.-M.K.); hadjipav@pharm.auth.gr (D.Η.-L.).; 2 Laboratory of Protein Chemistry, Proteomics and Epigenetic Signaling (PPES), Department of Biomedical Sciences, University of Antwerp, Wilrijk 2610, Belgium; E-Mail: ajay.palagani@ua.ac.be; 3 Laboratory of Eukaryotic Gene Expression and Signal Transduction (LEGEST), Department of Physiology, University of Ghent, Ghent 9000, Belgium; E-Mails: nadia.bougarne@vib-ugent.be (N.B.); Guy.Haegeman@UGent.be (G.H.)

**Keywords:** curcumin analogue, NF-κB, TNF, cancer, inflammation

## Abstract

In this study a series of curcumin analogues were evaluated for their ability to inhibit the activation of NF-κΒ, a transcription factor at the crossroads of cancer-inflammation. Our novel curcumin analogue **BAT3** was identified to be the most potent NF-κB inhibitor and EMSA assays clearly showed inhibition of NF-κB/DNA-binding in the presence of **BAT3**, in agreement with reporter gene results. Immunofluorescence experiments demonstrated that **BAT3** did not seem to prevent nuclear p65 translocation, so our novel analogue may interfere with NF-κB/DNA-binding or transactivation, independently of IKK2 regulation and NF-κB-translocation. Gene expression studies on endogenous NF-κB target genes revealed that **BAT3** significantly inhibited TNF-dependent transcription of IL6, MCP1 and A20 genes, whereas an NF-κB independent target gene heme oxygenase-1 remained unaffected. In conclusion, we demonstrate that **BAT3** seems to inhibit different cancer-related inflammatory targets in the NF-κB signaling pathway through a different mechanism in comparison to similar analogues, previously reported.

## 1. Introduction

In 1863, Rudolf Virchow was the first who made a connection between inflammation and cancer as he noticed the appearance of leucocytes in neoplastic tissues. Cancer is a multi-step disease [1] that affects people worldwide [2], and now it is well-established that is closely related to inflammation [3,4,5,6,7,8,9]. Various inflammatory mediators, such as chemokines and cytokines, are present in tumor cells, while targeting these, may provide new approaches for cancer therapy [10]. Cancer cells and tumor-associated immune cells contain multiple hyperactivated signal transduction pathways due to their transformation. Often they are activated following exposure to established cytotoxic therapies including ionizing radiation and chemical agents that cause DNA damage. Many pathways, activated in response to transformation or toxic stress, promote cell growth, invasion and prevent the processes of cell death. As a result of these findings, many drugs with varying specificities have been developed to block signaling by cell survival pathways in the hope of killing tumor cells and sensitizing them to toxic therapies [11]. Unfortunately, due to the plasticity of signaling processes, inhibition of any growth factor receptor or pathway has modest long-term effects on cell viability, tumor growth, and patient survival. As such the concept of multi-target treatment holds promise for cancer treatment or prevention [12,13,14]. As a result of this observation, there has been a growing interest in multifocal natural compounds, such as polyphenols, withanolides, xanthones, indanones, curcuminoids, which simultaneously inhibit multiple inter-linked signal transduction/survival pathways [11,15,16,17,18,19,20]. The past decades, researchers searching for new drugs to use in oncology have refocused on natural products [21,22].

A key player at the crossroad of cancer and inflammation is the transcription factor NF-κB, which is deregulated in various inflammatory diseases and cancer [6,23,24]. For example, NF-κB activity is strongly increased in estrogen receptor-negative, p53-mutated breast cancer cells such as MDA-MB231 [25,26,27]. NF-κB-regulated genes are involved in invasiveness, proliferation, angiogenesis, metastasis and inflammation. In cancer, Νuclear Factor kappa-light-chain-enhancer of activated B cells (NF-κB) is frequently autoactivated upon autocrine/paracrine production of pro-inflammatory cytokines and growth factors (*i.e*., TNF, EGF) [28,29]. In the “classic” NF-κB activation pathway, the heterodimer p50/p65 is inactive in the cytoplasm through its association with IκBα protein. After cellular activation by TNF (Tumor Necrosis Factor), IκBα is phosphorylated at two serine residues (Ser 32, Ser 36) by the IKK complex, containing the IκB kinases (IKKs): IKKα (also known as IKK1), IKKβ (also known as IKK2) and a non-catalytic regulatory subunit (NEMO/IKKγ) [30]. Activation of the IKK complex requires phosphorylation of two serine residues located in the “activation loop” within the kinase domain of at least one of the catalytic IKK subunits. The activated IKK complex phosphorylates in turn IκBα, causing its degradation by the proteasome and allowing free p50/p65 subunit to translocate into the nucleus in order to bind to κB sites in the promoter of several genes and to activate transcription. Besides regulation of NF-κB/DNA binding, additional pathways are involved in gene-specific NF-κB transactivation mechanisms depending on the epigenetic marks and cofactor repertoire recruited to the various NF-κB target sequences [31,32,33,34,35]. As such, various therapeutic strategies aim to decrease chronic hyperactivated NF-κB by pharmacological as well as phytomedicinal approaches [16,30,36,37,38,39,40,41,42,43].

Today, epidemiological and clinical studies with curcumin, a principal component of turmeric (a curry spice) showing strong pleiotropic anti-oxidant and anti-inflammatory activities and an excellent safety profile, reveal promising anti-cancer effects at the molecular level* in vitro* as well as* in vivo*. However, effects to prevent or reduce cancer so far did not achieve its optimum therapeutic outcome in past clinical trials, largely due to its low solubility and poor bioavailability [44,45]. As such, curcumin analogues with improved pharmacokinetic properties may enable its enhanced absorption and cellular uptake.

**Figure 1:**
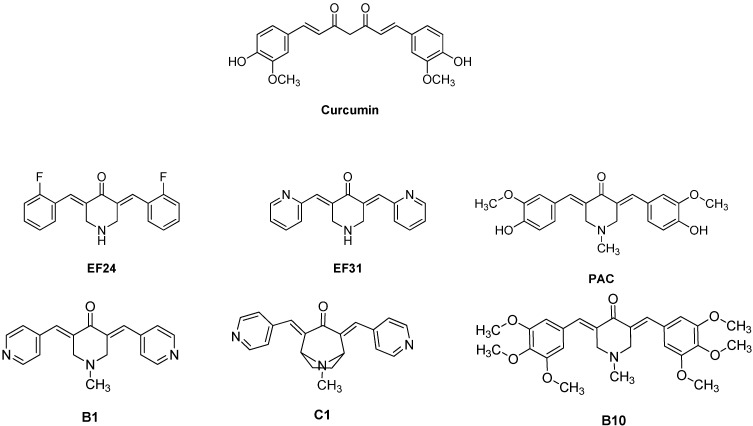
Chemical structures of curcumin and monoketone analogues of curcumin.

Curcumin or diferuloylmethane (Figure 1) is a polyphenolic yellow colored natural derivative, isolated from the dried rhizome of the herb *Curcuma longa Linn* (turmeric). It is a multi-target agent that interacts with multiple targets in the NF-κB signaling pathway implicated in cancer and inflammation [45,46,47,48,49,50]. Curcumin suppresses the activation of IKK, the phosphorylation and degradation of IκBα, nuclear translocation of the p65 subunit in several cancer cell lines as well as inhibition of the proteasome function [51,52,53,54,55,56,57,58]. Various curcumin analogues are also known to inhibit the NF-κB signal transduction pathway [59,60].

**Figure 2:**
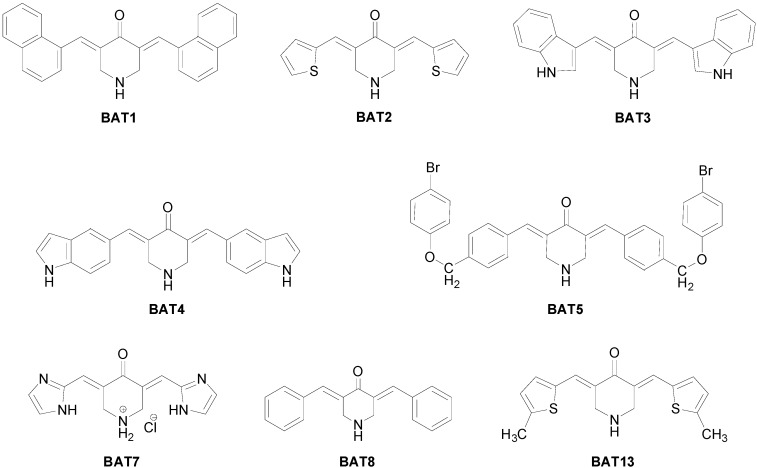
Chemical structures of the curcumin analogues investigated.

Recently we have published a series of curcumin analogues as anti-inflammatory and anti-proliferative agents. Among them analogue BAT3 presented interesting anti-proliferative activity with GI_50_ 3.3 uM in SF268 (Central Nervous System, glioma) cancer cell line [61]. Similarly, monoketone analogues of curcumin, EF24 and EF31 (Figure 1) show improved NF-κB inhibition, by attenuating the catalytic activity of IKK and blocking the nuclear translocation of NF-κB [62,63]. PAC (Figure 1) triggered apoptosis and inhibited several breast cancer-related proteins, among which NF-κΒ and its downstream effectors, such as cyclin D1 and Bcl-2 [64]. In addition, Yadav *et al*.; synthesized a series of heterocyclic cyclohexanone analogues of curcumin, which were examined for the ability to inhibit NF-κB transactivation in non-adherent K562 leukemia cells. Among these, the three analogues B1, B10 and C1 (Figure 1), showed potent cytotoxicity and inhibition of NF-κB activation [65]. In the present paper, we will evaluate the anti-inflammatory potencies of a series of novel curcumin analogues (Figure 2) [61], by means of NF-κB reporter gene assays, mRNA transcription analysis, NF-κB immunofluorescence microscopy and NF-κB/DNA binding studies.

## 2. Results and Discussion

### 2.1. Structure-Function Analysis of Anti-Inflammatory Activity of Curcumin Analogues by Means of NF-κB Reporter Gene Screening

**Figure 3:**
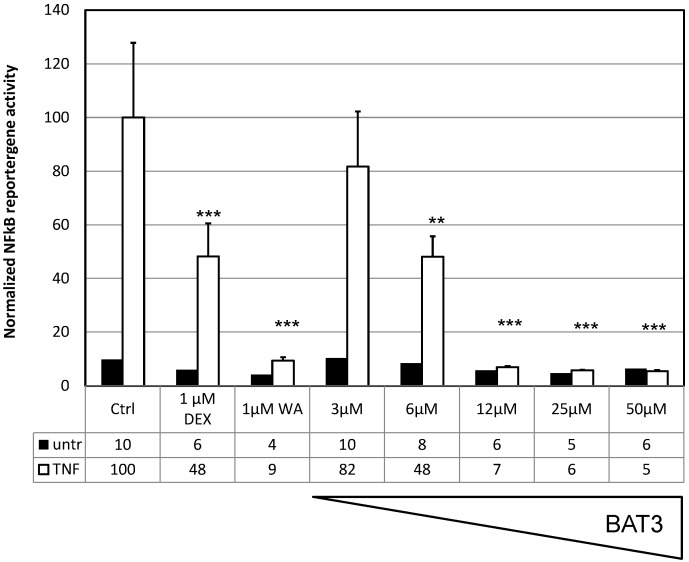
**BAT3** inhibits NF-κB-driven reporter gene expression. L929sA cells stably transfected with p(IL6κB)_3_50hu.IL6P-Luc and PGK-Gal were left untreated (untr) or were treated with 2000 IU/mL TNF for 4 h either alone or following a 24 h pretreatment with different doses of **BAT3**. Lysates were prepared for quantification of luciferase reporter gene expression levels and correction for potential cytotoxicity by evaluating corresponding constitutively expressed galactosidase levels. The relative induction factor is defined as the amount of luciferase produced in TNF-treated cells after normalization for galactosidase expression compared with the non-induced state. Statistical significant repression by **BAT3** is indicated as ** *p* < 0.01 or *** *p* < 0.001.

In order to investigate the potential NF-κB immunosuppressive effects of the various curcumin analogues, we performed dose response experiments in L929sA fibroblasts stably transfected with the recombinant NF-κB dependent reporter gene construct, p(IL6κB)_3_50hu.IL6P-Luc^+^, containing multiple NF-κB-responsive elements in front of a minimal IL-6 promoter, coupled to the luciferase reporter gene. Enhanced luciferase expression levels can be measured in response to TNF, as compared to the untreated setup, indicating that the cellular TNF response towards NF-κB activation is functional. Upon pretreatment with the various compounds, a significant dose-dependent inhibition of NF-κB reporter gene expression could be observed with analogues** BAT1**, **3** and **8**. **BAT3**, reveals the most potent NF-κB inhibition (Figure 3) with an approximate IC_50_ value of 6 µM, as compared to 15 µM for **BAT8** and 24 µM for **BAT1** (Table 1). Dexamethasone (**DEX**) and withaferin A (**WA**) were included as reference immunosuppressive compounds [19,66]. NF-κB-specific promoter effects are normalized against the constitutive promoter activity of the co-transfected PGK reporter gene plasmid.

**Table 1:** IC_50_ values of examined compounds.

Compounds	IC_50_ (µM)
**BAT1**	24
**BAT2**	inactive
**BAT3**	6.5
**BAT4**	inactive
**BAT5**	inactive
**BAT7**	inactive
**BAT8**	15
**BAT13**	inactive

### 2.2. Effect of the BAT3 Compound on NF-κB Activation, Translocation and NF-κΒ/DNA Binding

**Figure 4:**
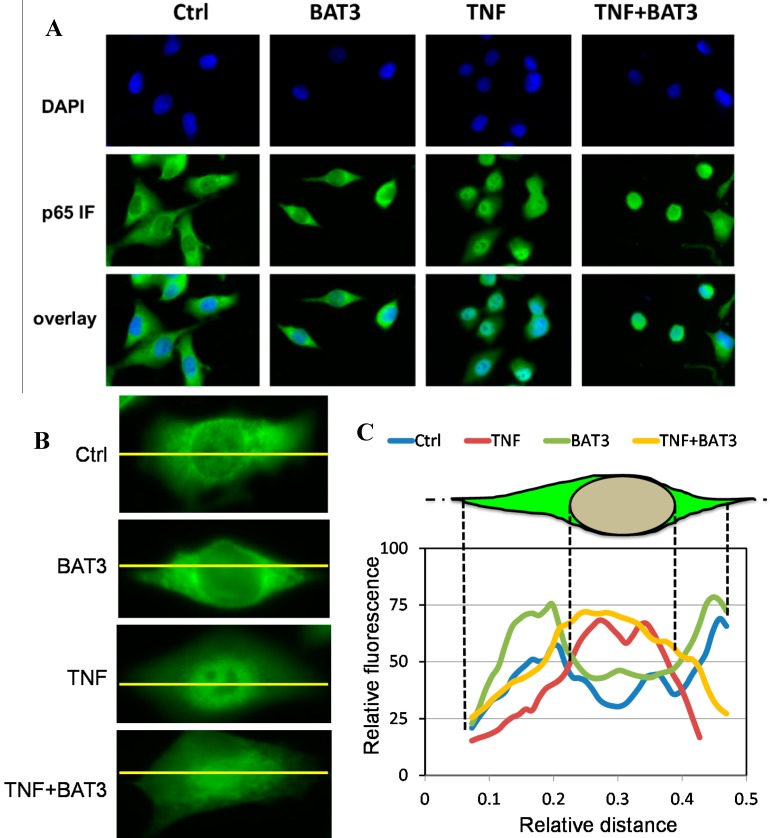
BAT3 does not inhibit nuclear NF-κB translocation. (**A**) A549 cells grown on coverslips were incubated with 25 µM **BAT3** for 2 h and then stimulated with 2000 IU/mL TNF for 30 min. After cell fixation, nuclei were visualized by blue DAPI staining and nuclear translocation of NF-κB was monitored by overlay of blue DAPI staining with anti-p65 green immunofluorescence microscopy; (**B**) Close-ups of NF-κB p65 immunofluorescence intensities across cytoplasmic and nuclear cell compartments for the difffeent treatments; (**C**) Cross-cellular (yellow line) fluorescence intensity plots were determined by Image J software (National Institutes of Health, Bethesda, MD, USA, version 1.49c, 2014) for the different treatments. For control and **BAT3** treated cells, p65 immunofluorescence can be observed predominantly in the cytoplasm, with a relative lower fluorescence intensity across the nuclear region as expected. However, upon evaluating immunofluorescence signals following TNF or combined TNF + **BAT3** treatment, p65 signal intensity is shifted more exclusively towards the nucleus as compared to the intensities observed in the cytoplasmic periphery.

Furthermore, upon immunofluorescent detection of subcellular pools of NF-κB p65 in cells treated with TNF alone or following combination treatment with **BAT3**, we could clearly detect TNF-induced translocation of NF-κB p65 from the cytoplasm to the nucleus (Figure 4). Remarkably, **BAT3** fails to block NF-κB translocation, since NF-κB p65 staining remains predominantly nuclear (Figure 4A–C). Also, since various NF-κB inhibitors sensitize for TNF incuded cell death, combination treatment of TNF + **BAT3** also revealed mild cytotoxic effects in immunofluorescence images [67,68]. Altogether, this suggests that NF-κB inhibition by **BAT3** cannot be explained by blocking TNF induced p65 nuclear translocation.

**Figure 5:**
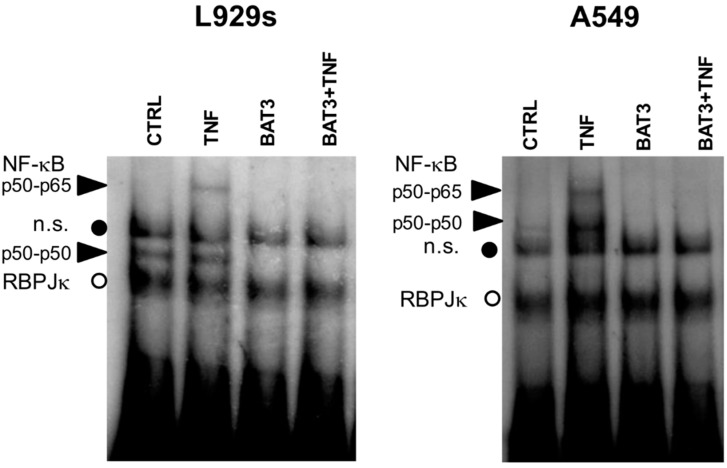
BAT3 inhibits NF-κB/DNA binding, L929sA and A549 cells were incubated with 25 µM BAT3 for 2 h and then stimulated with 2000 IU/mL TNF for 30 min. Corresponding nuclear lysates were incubated with a ^32^P-labeled IL-6κB site-containing probe and analyzed for NF-κB/DNA-binding complexes in EMSA. Loading of equal amounts of nuclear protein was verified by comparison with the constitutive binding activity of the repressor molecule recombination signal sequence-binding protein Jκ (RBP-Jκ). Black arrows indicate NF-κB binding complexes p50–p65 and p50–p50.

Next, we evaluated whether the inhibition of NF-κB reporter gene activity by **BAT3** occurs at the level of NF-κB/DNA-binding. Following cellular fractionation [69], we measured TNF-induced NF-κB/DNA binding levels in EMSA following 30 min TNF treatment, either alone or following 2 h pretreatment with **BAT3** in L929 fibrosarcoma and A549 lung epithelial cells (Figure 5). As expected, TNF stimulation induces a significant increase in NF-κB/DNA binding. Interestingly, a 2 h pretreatment of cells with **BAT3** prevents basal and/or TNF-induced NF-κB/DNA binding. Of special note, the binding levels of a constitutive nuclear recombination signal sequence-binding protein Jκ (RBP-Jκ), binding to the same NF-κB DNA motif remain unaffected under all conditions tested, pointing to a specific inhibition of nuclear NF-κB/DNA binding [69]. These results are in line with the reporter gene results.

### 2.3. Transcriptional Effect of the BAT3 Compound on Endogenous NF-κB Target Genes

**Figure 6:**
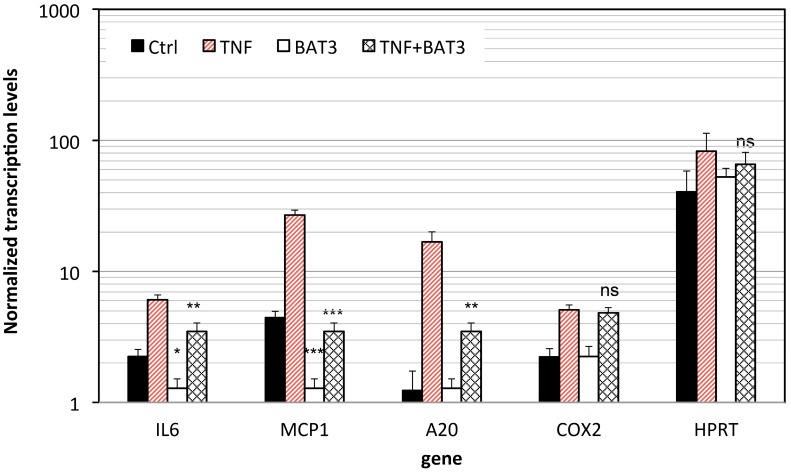
BAT3 inhibits endogenous NF-κB target gene expression. L929sA cells left untreated or treated with 25 µM BAT3 for 2 h were subsequently stimulated with 2000 IU/mL TNF for 3 h. Total cytoplasmic RNA was isolated, and converted to cDNA. Corresponding gene expression levels of TNF inducible genes IL6, IL8, A20, COX2 and the constitutively transcribed HPRT (Hypoxanthine-guanine phosphoribosyltransferase 1) were evaluated by Sybr green Q-PCR analysis and were normalized for gene expression of the housekeeping gene β-actin. Statistical significant repression by BAT3 is indicated as not significant *ns p* > 0.05, * *p* < 0.05, ** *p* < 0.01 or *** *p* < 0.001.

Next, NF-κB-suppressive effects of the **BAT3** compound were validated by real-time QPCR on the expression of different NF-κB target genes involved in inflammation and prevention of apoptosis. The relative amount of target mRNA was determined using the comparative threshold (Ct) method by normalizing target mRNA Ct values to those for β-actin. The results show that compound **BAT3** significantly downregulates the expression of various TNF inducible NF-κB target genes such as IL-6, MCP1 and A20. Surprisingly, another TNF inducible NF-κB target gene COX2 remains unaffected in presence of **BAT3**. Also, the constitutively expressed target gene Hypoxanthine-guanine phosphoribosyltransferase (HPRT), which does not contain an NF-κB binding motif in its promoter and as such does not respond to TNF, remains unaffected by **BAT3** treatment (Figure 6). Of special note, in contrast to general inhibition of NF-κB/DNA binding by **BAT3** observed in synthetic reportergene (Figure 3) and* in vitro in vitro* EMSA DNA-binding studies (Figure 5), evaluation of **BAT3** effects at endogenous NF-κB target genes reveals a more selective mechanism of NF-κB inhibition. Indeed, combinatorial control of transcription factor binding and chromatin dynamics by posttranslational modifications may further contribute in selective nuclear regulation of NF-κB-target genes by **BAT3** [31,70]. Along the same line, we have previously demonstrated selective inhibition of endogenous NF-κB target genes by MSK1 and MAPK inhibitors via coregulation of transcription factor and chromatin histone marks [35,71]. As such our results suggest that **BAT3** may interfere more selectively with NF-κB dependent gene expression at the chromatin-DNA interface. In line with our hypothesis, various reports have recently demonstrated significant effects of curcumin analogues on histone acetylation of inflammatory genes [72,73]. Whether curcumin analogues may interfere with MSK1 regulation of NF-κB-chromatin marks needs further investigation.

## 3. Experimental Section

### 3.1. Compounds, Cytokine and Reagents

The curcumin analogues **BAT** (Figure 2) were prepared as previously described [61]. Withaferin A was purchased from Chromadex (Irvine, CA, USA) and dexamethasone (DEX) was obtained from Sigma-Aldrich (St. Louis, MO, USA). Compounds were stored as 10 mM solutions in DMSO at −20 °C. A549 cells were purchased from ATCC. Recombinant murine TNF, produced in *Escherichia coli* and purified to at least 99% homogeneity, had a specific biological activity of 8.58 × 10^7^ IU/mL of protein as determined in a standard TNF cytolysis assay [35]. Reference TNF (code 88/532) was obtained from the National Institute of Biological Standards and Control (Potters Bar, UK).

### 3.2. Transfection Procedure—Cell Cultures

The murine fibrosarcoma L929A cells, stably transfected with the recombinant NF-κB-driven reporter gene construct p(IL6κB)_3_50hu.IL6P-Luc^+^ and co-transfected with the reference reporter plasmid PGK-neogalactosidase, which constitutively expresses a neomycin resistance protein fused to a galactosidase reporter protein controlled by the housekeeping promoter phosphoglycerate kinase (PGK), were previously described [33,35]. L929sA and A549 (human lung epithelial) cells were grown at 37 °C, 95%–98% humidity and 5% CO_2_ incubator in Dulbecco Modified Eagle’s Medium (DMEM) (Gibco, Invitrogen, Carlsbad, CA, USA), supplied with 5% fetal calf serum, 5% Newborn calf serum (Greiner bio-one, Frickenhausen, Germany), 0.2% of P/S antibiotic (100 U/mL penicillin, 0.1 mg/mL streptomycin) (Gibco, Invitrogen) and 10% glutamine 2 mM.

### 3.3. Treatment—Cell Lysis

Twenty four hours before treatment with the compounds, L929 cells were seeded in 96-well plates such that they were confluent at the time of the experiment. After cells were treated with the compounds overnight, cells were left untreated or treated with 2500 U/mL TNF for 4 h. After the indicated induction time, medium supernatant was removed, the cells were washed with ice-cold PBSA and lysed in reporter lysis buffer (Promega Biotec, Madison, WI, USA).

### 3.4. Reporter Gene Analysis

Analysis—Luciferase assays were carried out according to the instructions of the manufacturer (Promega Biotec) and have been described previously [35]. Light emission was measured in a luminescence microplate counter (Top-Count; Packard Instrument Co., Meriden, CT, USA). Normalization of luciferase activity, expressed in arbitrary light units, was performed by measurement of β-galactosidase levels in a chemiluminescent reporter assay Galacto-Light kit (Tropix, Bedford, MA, USA).

### 3.5. Immunofluorescence Microscopy

Lung epithelial cells (A549) were seeded on coverslips and starved in phenol red- and serum-free medium for 24 h before starting treatment with the compounds and inductions. Cells were pretreated with the compounds of interest (25 μM) for 2 h, followed by TNF induction for 30 min. The cells were fixed in 4% formaldehyde in phosphate-buffered saline (PBS), permeabilized with ice cold aceton for 3 min, and then blocked in 5% milk–10% FCS–0.3% bovine serum albumin–0.3% Triton X-100 in PBS for 1 h at room temperature. P65 was visualized by using a 1:200 dilution of the anti-p65 antibody overnight (NF-κB p65 (C20) rabbit polyclonal 200 µg/mL Santa Cruz Biotechnology (Santa Cruz, CA, USA), followed by probing with a 1:800 dilution of Alexa Fluor 488 donkey anti rabbit IgG (H+L) 2 mg/mL (Invitrogen, Carlsbad, CA, USA). Cell nuclei were visualized by DAPI staining. Samples were analyzed by a Zeiss axiovert 200 using AxioVision Rel.4.5 software (Carl Zeiss, Jena, Germany). Densitometric line plot profiles of immunofluorescencen intensities across cytoplasmic and nuclear cell regions were determined by ImageJ freeware (National Institute of Health, Bethesda, MD, USA).

### 3.6. Electrophoretic Mobility Shift Assay (EMSA)

L929sA and A549 were seeded in 6-well plates at 3 × 10^5^ cells/well and treated 2 h with the compound of interest (25 μM) and 30 min with or without TNF. After treatment, cells were washed with ice-cold PBS and pelleted in 1 mL PBS by centrifugation for 10 min at 2600 rpm (4 °C). Preparation of nuclear extracts has been described previously [69]. For EMSA, equal amounts of protein were incubated for 25 min with an NF-κB-specific ^32^P-labeled oligonucleotide and binding mix as described previously [19]. For supershift assay, antibodies were preincubated to the sample of interest for 10 min prior to incubation with radiolabeled probe [69]. Labeling of the oligonucleotides was performed with [α-^32^P]-dCTP by using Klenow enzyme (Boehringer, Mannheim, Germany). For EMSA competition assays, 100 fold excess of unlabeled NF-κB oligonucleotide was added to the binding mix. The NF-κB oligonucleotide comprises the sequence: 5'-AGCTATGTGGGTTTTCCCATGAGC-3', in which the single IL6 promoter-derived NF-κB motif is underlined. Samples were loaded on a 6% polyacrylamide gel run in 0.5× TBE buffer (pH 8) and complexes formed were analyzed using Phosphor Imager Technology [74].

### 3.7. RNA Isolation and Real-Time Q-PCR Analysis

Murine fibrosarcoma L929A were seeded in 6-well plates 24 h before treatment with the compounds, such that they were confluent at the time of the experiment. Cells were pretreated with the compounds of interest (25 μM) for 2 h, followed by TNF induction for 3 h. Total RNA was extracted with TRIzol (Invitrogen, Merelbeke, Belgium), as described by the manufacturer. The quality and the quantity of the RNA were measured by Nanodrop equipment (Thermo Fisher Scientific, Waltham, MA, USA). Concentrations of samples were determined and 500 ng RNA was used in a RT-step with MMLV reverse transcriptase (Promega, Madison, WI, USA) to produce the respective cDNA. Briefly an aliquot of 500 ng of RNA sample, 1 μL oligo (dT) (Promega), 10 μL MMLV buffer 5× (Promega), 5 μL 2.5 mM dNTP mix, 0.5 μL RNasin^®^, 1 μL mMLV reverse transcriptase (Promega) were mixed and adjusted to a final volume of 50 μL by DEPC-H_2_O in a PCR tube, and the reverse transcription reaction was conducted using the following conditions: 60 min at 42 °C, 15 min at 75 °C, and 30 min at 4 °C in a PCR machine (Perkin Elmer, Waltham, MA, USA).

The Q-RT-PCR was performed using Invitrogen Sybr green platinum Supermix-UDG on a iCycler apparatus (Bio-Rad, Eke, Belgium). The obtained cDNA was diluted 5 times before use. Briefly 7.5 μL SYBR Green master mix, 0.75 μL FW primer (5 μM, diluted in baxter H_2_O), 0.75 μL RV primer (5 μM, diluted in Baxter H_2_O) and 1 μL QPCR- H_2_O (Baxter H_2_O) were added to 5 μL of the diluted cDNA in a Q-PCR plate. Primer specificity was verified by melting curve analysis. Primer efficiencies were analyzed by amplification of a cDNA standard dilution. All amplifications were performed in triplicate and data were analyzed using Genex software (Bio-Rad). β-actin was used as housekeeping gene for normalization of the various target genes. Results are expressed as relative gene expression,* i.e*., N-fold differences in target gene expression relative to the β-actin gene, were determined as N *target* = 2^Δ*Ct sample*^, where the Δ*Ct* (cycle threshold) value of the sample was determined by subtracting the average *Ct* value of the target gene from the average *Ct* value of β-actin gene (primer efficiency of 100%). Q-PCR primers are summarized in Table 2.

**Table 2:** Primers sequences used in the real-time Q-PCR (FW: forward; RV: reverse).

Primers	Sequence
IL6 FW	GTCCTTCCTACCCCAATTTCC
IL6 RV	TTGGTCCTTAGCCACTCCTTC
A20 FW	AACCAATGGTGATGGAAACTG
A20 RV	GTTGTCCCATTCGTCATTCC
HO1 FW	AAGACCGCCTTCCTGCTCAAC
HO1 RV	CGAAGTGACGCCATCTGTGAGG
COX2 FW	TGTGCAAGATCCACAGCCTA
COX2 RV	TCTGGAGTGGGAGGCACTT
β-actin FW	CTTCTAGGCGGACTGTTACTGA
β-actin RV	CCATGCCAATGTTGTCTCTTAT
MCP1 FW	TCC CTG GTC CAA AGG TTT TTC
MCP1 RV	CTT GGT TTC CCC ATT GGA TCT
HPRT1 FW	CCTAAGATGAGCGCAAGTTGAA
HPRT1 RV	CCACAGGACTAGAACACCTGCTAA

### 3.8. Statistical Analysis

Statistics were performed using one way anova followed by Dunnett’s post-test and unpaired student *t*-test using Graph Pad Prism5 software.

## 4. Conclusions

Previous reports have shown that curcumin analogues inhibit NF-κΒ transcriptional activity by blocking general activation and nuclear translocation of NF-κB [62,63]. Interestingly, our results reveal that the novel analogue BAT3 selectively inhibits ΝF-κΒ-dependent gene expression, most presumably through gene specific effects of NF-κB binding to chromatin-DNA [33,35,71]. Altogether we demonstrate that the BAT3 curcumin analogue holds promise to selectively suppress the NF-κB signaling pathway. As such, genomewide investigation of BAT3 effects at the NF-κB-chromatin interface by chromatin immunoprecipitation sequencing experiments in different cell types may further corroborate its selective immunomodulatory properties in cancer-inflammation. Finally,* in vivo* studies with BAT3 are required to further evaluate its bioavailability and therapeutic efficacy, as well as potential improvement of its pharmacokinetic properties in comparison to curcumin.
